# Thoracoscopic Implantation of Epicardial Left Ventricular Lead for Cardiac Resynchronization Therapy

**DOI:** 10.3390/jcdd9050160

**Published:** 2022-05-16

**Authors:** Hye Ree Kim, Kyunghee Lim, Seung-Jung Park, Jong-Sung Park, Ju Youn Kim, Suryeun Chung, Dong-Seop Jung, Kyoung-Min Park, Young Keun On, June Soo Kim

**Affiliations:** 1 Division of Cardiology, Department of Internal Medicine, Gyeongsang National University Hospital, Gyeongsang National University School of Medicine, Jinju 52728, Korea; hrmanse@naver.com; 2 Division of Cardiology, Department of Internal Medicine, Dong-A University College of Medicine, Busan 49201, Korea; jinjinsama@naver.com (K.L.); thinkmed@naver.com (J.-S.P.); 3 Division of Cardiology, Department of Internal Medicine, Heart Vascular and Stroke Institute, Samsung Medical Center, Sungkyunkwan University School of Medicine, Seoul 06351, Korea; kzzoo921@gmail.com (J.Y.K.); bkm1101@hanmail.net (K.-M.P.); yk.on@samsung.com (Y.K.O.); js58.kim@samsung.com (J.S.K.); 4 Department of Thoracic and Cardiovascular Surgery, Samsung Medical Center, Sungkyunkwan University School of Medicine, Seoul 06351, Korea; suryeun.chung@samsung.com (S.C.); opheart1@gmail.com (D.-S.J.)

**Keywords:** epicardial, thoracoscopy, cardiac resynchronization therapy, heart failure

## Abstract

(1) Background: Limited data exist on the safety and efficacy of epicardial left ventricular (LV) lead placement using video-assisted thoracoscopic surgery (VATS) for cardiac resynchronization therapy (CRT). (2) Methods: Acute and post-discharge outcomes of CRT were compared between patients with epicardial LV leads (Epicardial-LV group, *n* = 13) and those with endocardial LV leads (Endocardial-LV group, *n* = 243). (3) Results: Epicardial LV leads were implanted via VATS alone (*n* = 8) or along with mini-thoracotomy (*n* = 5), for failed endocardial implantation (*n* = 11) or recurrent lead dislodgement (*n* = 2). All epicardial procedures under general anesthesia with one-lung ventilation were successfully completed in 1.0 ± 0.4 h without phrenic nerve stimulation. LV pacing thresholds in the epicardial-LV (1.5 ± 1.0 V) and endocardial-LV (1.3 ± 0.8 V) were comparable (*p* = 0.651). All patients were discharged alive post-VATS 8.8 ± 3.9 days. During the follow-up (34.3 ± 28.6 months), all patients with epicardial LV leads stayed alive except for one cardiac death post-CRT 14 months and one heart transplantation post-CRT 30 months. All epicardial LV leads maintained stable performance without dislodgement/significant changes in pacing threshold/impedance. LV lead dislodgement occurred only in endocardial-LV (7/243, 2.9%). Efficacy in both groups was comparable in terms of QRS narrowing, increase in LV ejection fraction, and survival free of cardiac death, or heart-failure-related hospitalization. (4) Conclusions: Epicardial LV lead placement using VATS can be a safe and effective alternative to endocardial implantation, with comparable acute and post-discharge outcomes achieved by both approaches.

## 1. Introduction

Cardiac resynchronization therapy (CRT) in the form of biventricular stimulation decreases mortality and morbidity in selected patients with symptomatic heart failure (HF) characterized by interventricular conduction delay [1,2,3,4,5]. Conventional CRT recipients receive transvenous leads into their right atrium (RA), right ventricle (RV), and cardiac veins on the left ventricular (LV) free wall. Despite recent technological advancements in endocardial LV leads and delivery tools, the failure rate of LV lead implantation is still high (5–10%) for various reasons, including unsuitable cardiac vein anatomy, high pacing threshold, or phrenic nerve stimulation (PNS) [6,7]. For these clinical situations, epicardial LV lead placement can be an alternative or rescue option [8,9,10].

In our center, a minimally invasive video-assisted thoracoscopic surgery (VATS) began to be actively utilized in March 2010 for epicardial ablation of atrial fibrillation or implantation of LV leads, avoiding more invasive median sternotomy or full thoracotomy [8,11,12]. In the present study, we sought to evaluate the acute and post-discharge safety and effectiveness of epicardial LV lead implantation using VATS. In addition, we compared overall CRT outcomes with epicardial LV leads to those with conventional endocardial LV leads.

## 2. Materials and Methods

### 2.1. Patient Population

**Figure 1:**
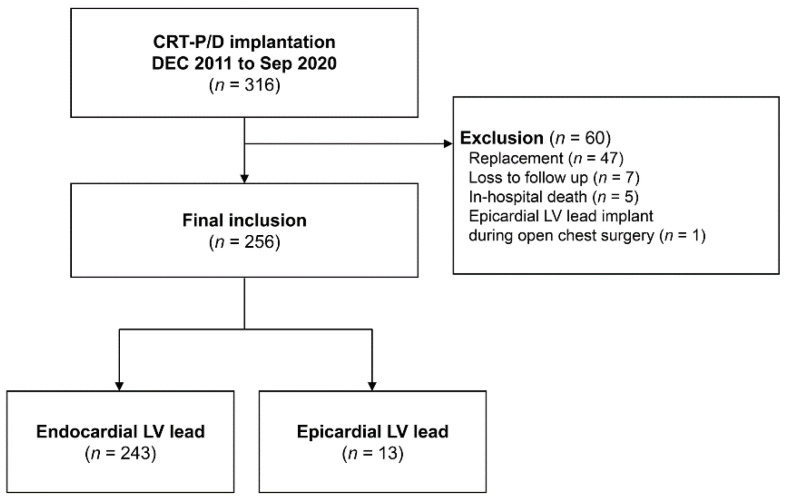
Flow diagram of patient enrollment. CRT-P/D = cardiac resynchronization therapy-pacemaker/defibrillator, LV = left ventricle.

From October 2005, we prospectively entered information into our registry of cardiac implantable electronic devices on demographic, clinical, and device-related parameters in conjunction with electrocardiographic and echocardiographic data for all patients undergoing CRT implantation at our institution. In the registry, we carefully searched for consecutive patients with epicardial LV lead placement (Epicardial group). As a control group, data on consecutive patients who received successful transvenous LV lead placement during the same study period were also obtained (Endocardial group). Exclusion criteria were (1) replacement of CRT device, (2) epicardial LV implantation during open chest surgery, (3) in-hospital death, and (4) loss to follow-up after CRT implantation (Figure 1). All patients in both groups had New York Heart Association (NYHA) functional class II−IV HF symptoms refractory to optimal medical therapy (at least for 3 months), severe LV systolic dysfunction defined as LV ejection fraction (LVEF) ≤35%, and dyssynchrony suggested by wide QRS complexes (>120 msec). This study complied with the Declaration of Helsinki, and Samsung medical center institutional review board (IRB) approved the study protocol and waived the need for consent from patients or relatives (IRB No. 2020-09-014).

### 2.2. Epicardial LV Lead Implantation for CRT

**Figure 2:**
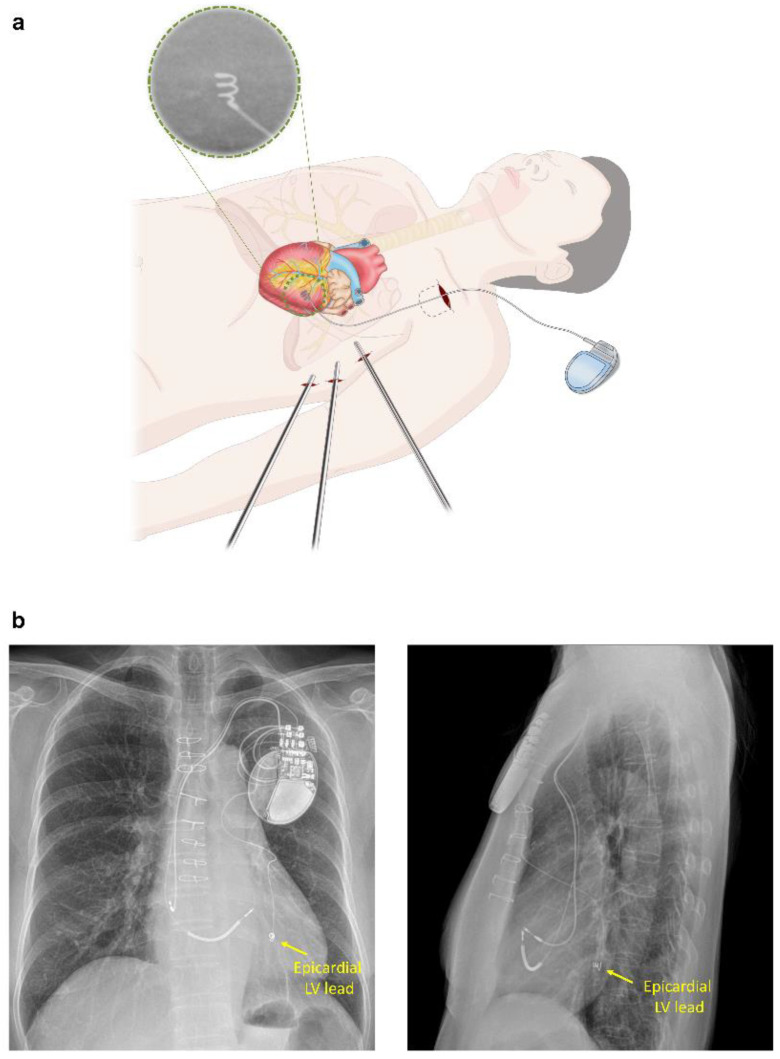
(**a**) Detailed illustration of epicardial LV lead implantation using the thoracoscopic approach; (**b**) post-operative chest X-ray showing an epicardial LV lead implanted into the LV lateral wall (Patient No.11). LV = left ventricle.

Epicardial LV lead implantation using VATS was performed as a second stage procedure after RV and RA leads were implanted in the electrophysiology laboratory. Under general anesthesia, using double endoluminal intubation for selective lung ventilation, patients were placed in the right lateral decubitus position with the left chest elevated 30–40°. The left subclavian generator pocket created during the previous endocardial approach was reopened before one-lung ventilation. A 2 cm incision for a 15 mm port was made in the sixth left intercostal space at the posterior axillary line. A 30° thoracoscope was inserted through the 15 mm port for inspection of the left thoracic cavity. Two additional 5 mm ports were positioned in the fourth left intercostal space anterior to the anterior axillary line for the second port and mid-axillary level for the third port for introducing thoracoscopic grasp and dissector, respectively (Figure 2a). In cases of pleural adhesion, a 3 to 4 cm long left mini-thoracotomy was performed through the fourth intercostal space between the anterior and mid-axillary line. A 2 cm pericardiotomy was performed anterior to the phrenic nerve to expose the LV lateral wall. After moving the thoracoscope from the 15 mm port to the third port, a screw-in unipolar pacing lead (Medtronic Model 5071 Pacing lead, Minneapolis, MN, USA) was implanted via the 15 mm port into the LV lateral wall around an obtuse marginal coronary artery. After threshold measurements, the proximal end of the LV lead was tunneled up to the subclavian pocket and connected to the generator along with the previously implanted RV and RA leads. A chest tube was inserted through the third (mid-axillary) port. After completion of the operation, the location of the epicardial LV lead was confirmed by chest X-ray (Figure 2b). The procedure time for LV lead implantation was estimated from the initiation of one-lung ventilation (just before thoracoscope insertion) to the resumption of two-lung ventilation (after completion of LV lead implantation).

### 2.3. Acute and Post-Discharge Outcomes

To assess in-hospital outcomes in the Epicardial group, we evaluated procedure time, the success rate of implantation, immediate QRS narrowing, any change in NYHA functional class, post-implant duration of care at intensive care unit (ICU), length of post-implant hospital stay, mortality, and procedure-related complications (LV lead dislodgement, the presence of PNS, hemothorax, or lead/device infection).

After discharge, clinical follow-ups with CRT-device interrogations were performed approximately 2 weeks after surgery and then every 3 months or when clinically indicated at our dedicated device clinic. Chest X-ray, 12-lead electrocardiograms, and biventricular pacing percentage (BiV-*p*%) reports were obtained at each clinic visit. The BiV-*p*% was calculated by averaging the values obtained at 3 months, 12 months, and the last visit after the procedures. During the follow-up period, adjustments to CRT pacing parameters such as pacing rate, mode, and atrioventricular/ventriculoventricular delay were left to the physician’s discretion.

The long-term efficacy and safety of epicardial LV leads were evaluated and compared with those of endocardial LV leads in terms of changes in pacing threshold and impedance. Delayed-onset lead-related complications were also carefully reviewed. Clinical responses to CRT such as LV reverse remodeling, overall and cardiac mortality, and HF-related hospitalization (HF hospitalization) were compared between the Epicardial and Endocardial groups. Pre- and post-operative assessments of LVEF, LV end-diastolic volume (EDV), and LV end-systolic volume (ESV) were obtained using commercially available equipment (Vivid 9 or 7 from GE Healthcare, Chicago, IL, USA; Sonos 5500 from Philips, Andover, MA, USA). All death cases were defined as cardiac origin unless a definite non-cardiac cause could be identified. HF hospitalization was confirmed according to the 2016 European Society of Cardiology guidelines following careful assessment of HF symptoms or signs, chest radiography, echocardiography, and biomarkers.

### 2.4. Statistical Analysis

Baseline data are presented as frequencies or the mean ± standard deviation (SD). Categorical variables were compared using χ2 tests. Comparisons of continuous variables in the same patients were performed using Wilcoxon signed-rank test or paired *t*-test when appropriate. Continuous variables between the Epicardial and Endocardial groups were compared using the Mann–Whitney test. Relative change in QRS duration (QRSd), LV volumes, and LV lead pacing variables were calculated as the differences between the last follow-up values and the baseline values divided by the baseline values. The probability of freedom from events was calculated according to the Kaplan–Meier method. A *p*-value < 0.05 was considered to indicate statistical significance. All statistical analyses were performed using SPSS version 25.0 (SPSS, Chicago, IL, USA).

## 3. Results

### 3.1. Baseline Characteristics

From December 2011 to September 2020, we identified 13 consecutive patients who underwent VATS epicardial LV lead implantation after the failure of the percutaneous approach due to the absence of suitable lateral cardiac vein (*n* = 5), tortuous coronary sinus and/or target branches (*n* = 3), anomalous cardiac veins (*n* = 3), or recurrent dislodgement of previous LV lead (*n* = 2). The mean (±SD) age of the 13 patients was 61.9 ± 8.9 years, and male patients accounted for 46% (Table 1). An NYHA class III−IV HF symptom was observed in 62% (8/13) of patients. Baseline LVEF and QRSd were 26.4 ± 4.6% and 190 ± 31 ms, respectively. About one-third of patients (31%, 4/13) had ischemic cardiomyopathy as their HF etiology. Previously, six patients had undergone sternotomy, including coronary artery bypass grafting (CABG) surgery (*n* = 2), valve replacement (*n* = 2), CABG with valve replacement (*n* = 1), and closure of ventricular septal defect (*n* = 1).

During the same period, 243 consecutive patients in the Endocardial group with de novo CRT implantation were found in our CRT registry. There was no significant difference between the two groups in terms of baseline demographics, electro- and echocardiographic variables, CRT device-related factors, and discharge medications except for a wider pre-CRT QRSd (190 ± 31 vs. 170 ± 36 ms, *p* = 0.019) and more frequent previous cardiac surgery (38.5% vs. 17.3%, *p* = 0.055) in the Epicardial group (Table 2).

### 3.2. Acute Outcomes after Epicardial LV Lead Implantation for CRT

All epicardial implantations of LV leads were successfully completed via VATS alone (*n* = 8) or along with mini-thoracotomy (*n* = 5) without significant immediate post-procedural complications. Mini-thoracotomy was needed in five patients with pleural adhesions. Of the five patients, three had a previous cardiac surgery such as CABG or ventricular septal defect closure. General anesthesia with one-lung ventilation was well tolerated by all patients. The mean (±SD) procedure time was 1.0 ± 0.4 h. The immediate post-implant LV pacing threshold was 1.5 ± 1.0 V (at 0.5 ± 0.1 ms of pulse width), which was not significantly different from that of the Endocardial group (1.3 ± 0.8 V at 0.5 ± 0.1 ms, *p* = 0.651) (Table 2). All patients in the Epicardial group were successfully transferred to the general ward after short-term care in the ICU (18.5 ± 16.6 h). Chest tubes were removed in 3.1 ± 1.6 days, and all patients were discharged after a post-CRT hospital stay of 8.8 ± 3.9 days with a significant symptom improvement (NYHA functional class, 3.2 ± 0.7 to 2.0 ± 0.6, *p* = 0.001). There was no PNS, hemothorax, or lead dislodgement during the index hospitalization.

### 3.3. Post-Discharge Outcomes

**Figure 3:**
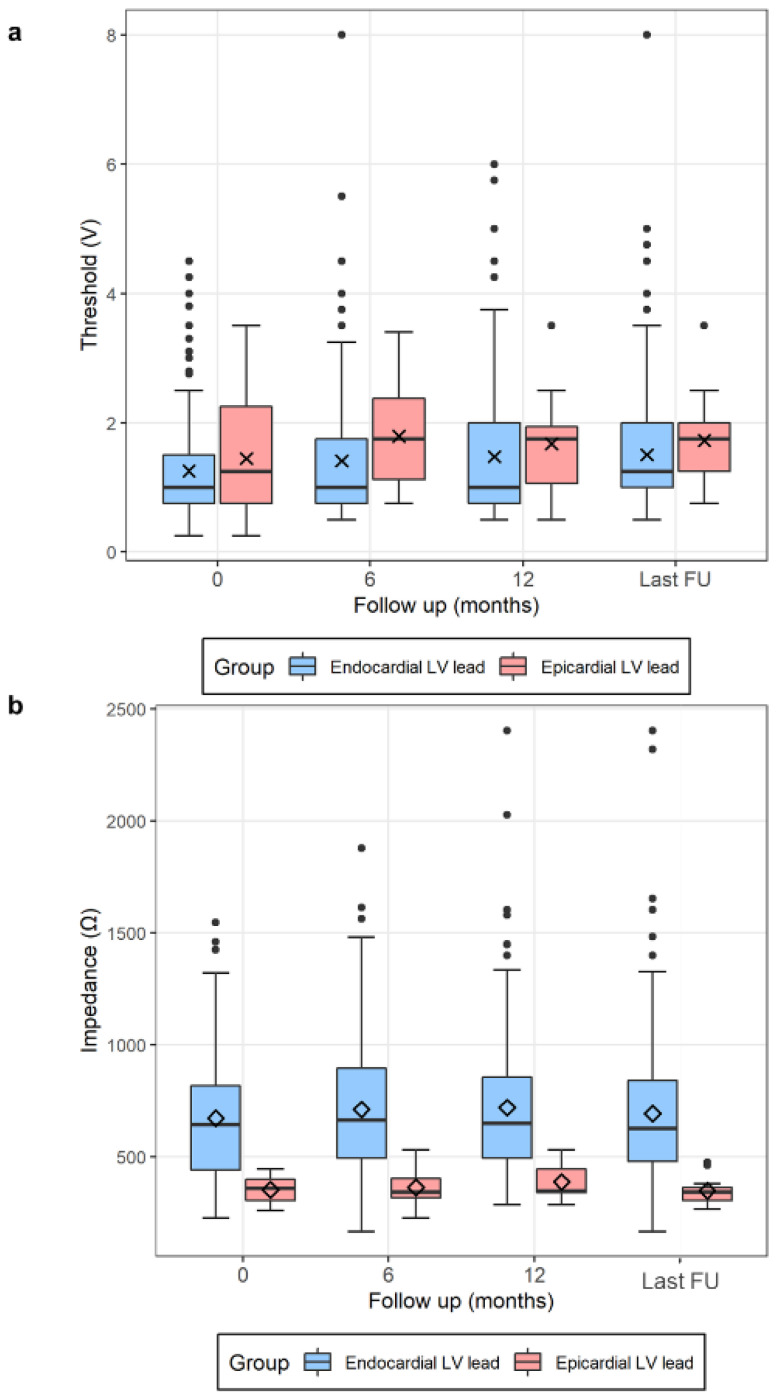
Serial changes in pacing threshold (**a**) and impedance (**b**) of epicardial and endocardia LV leads during the follow-up. LV = left ventricle.

*Post-discharge* performance of epicardial LV leads was evaluated and compared with that of endocardial LV leads (Table 3 and Supplementary Table S1). The Epicardial group showed no significant change in the LV pacing threshold (1.5 ± 1.0 to 1.7 ± 0.7, *p* = 0.154) and impedance (353 ± 51 to 349 ± 62, *p* = 0.859) during the 34.3 ± 28.6 months of follow-up (Figure 3). In addition, the relative changes in LV pacing threshold and impedance in both groups were not significantly different from each other (Table 3 and Table S1). In the Endocardial group, late LV lead dislodgement was found in 7 (2.9%) of 243 patients. However, there were no late epicardial-LV-lead-related complications such as dislodgement, PNS, lead fracture, hemopericardium, or hemothorax during 34.3 ± 28.6 months of follow-up.

The efficacy of CRT with epicardial versus endocardial leads was also compared during the post-discharge 34.3 ± 28.6 months of follow-up. Patients in the two groups revealed a marked and comparable QRS narrowing. Additionally, a remarkable LV reverse remodeling was observed in terms of LVEF increase and LVESV reduction. The LVEF increased from 25.7 ± 3.9% to 37.7 ± 15.1% in the Epicardial LV group (*p* = 0.023), while it increased from 25.6 ± 6.2% to 38.1 ± 14.5% in the Endocardial LV group (*p* < 0.001).

**Figure 4:**
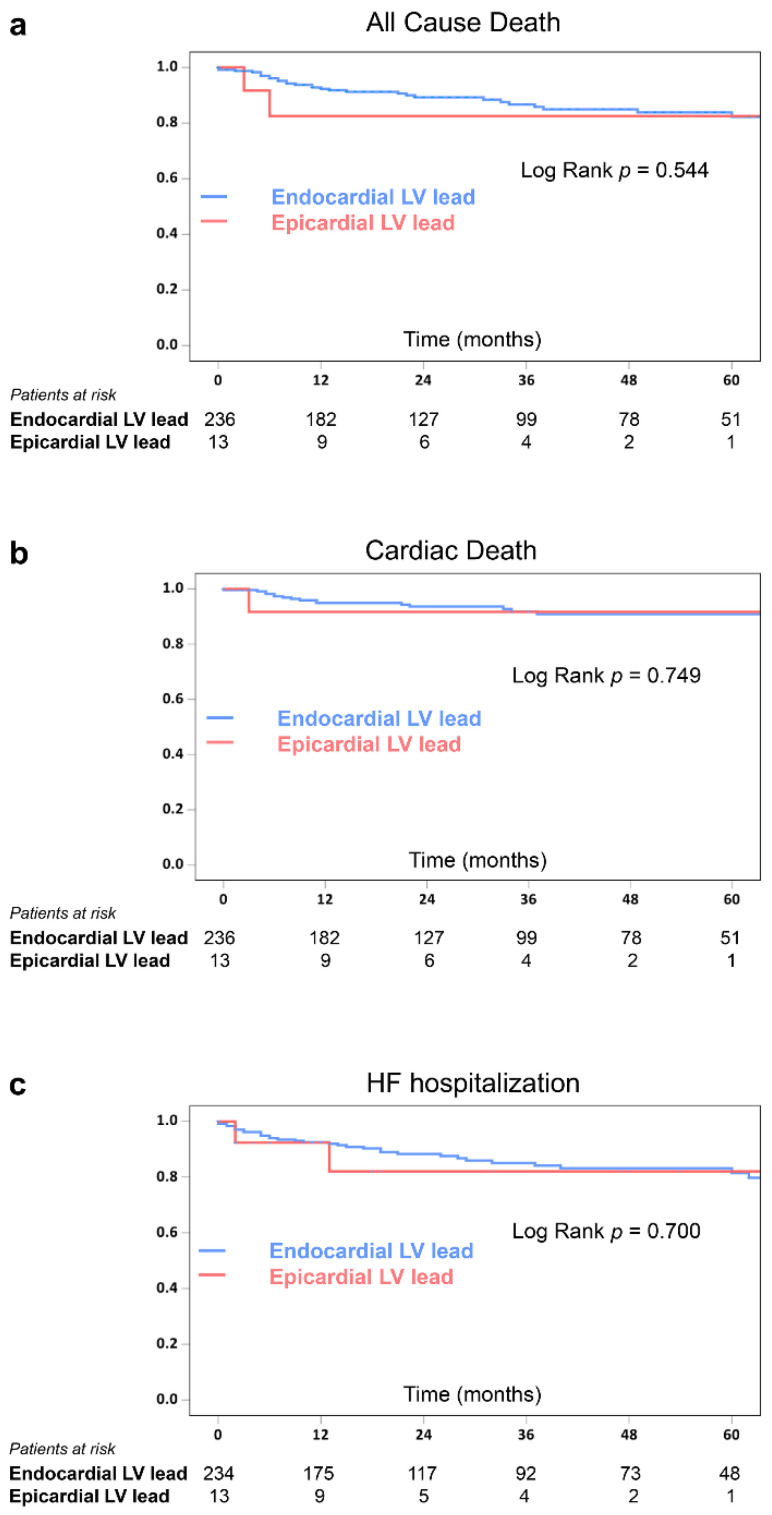
Kaplan–Meier survival curves of time to all-cause death (**a**), cardiac death (**b**), and HF hospitalization (**c**) in the Endocardial and Epicardial groups. HF = heart failure, LV = left ventricle.

All patients in the Epicardial group stayed alive except for one patient who died post-CRT 14 months and one heart transplantation patient who died post-CRT 30 months. The survival rates free of all-cause death (*p* = 0.544), cardiac death including left ventricular assist device implant and heart transplantation (*p* = 0.749), and HF hospitalization (*p* = 0.700) were comparable between the two groups (Figure 4).

## 4. Discussion

The present study showed that the VATS epicardial LV lead implantation was safe and effective in patients with previous implantation failure or late dislodgement of the endocardial LV lead. Moreover, CRT with epicardial versus endocardial LV lead was comparable in overall LV lead performance, QRS narrowing, echocardiographic reverse remodeling, and clinical outcomes.

The failure rate of endocardial LV lead implantation has been progressively decreasing because of the advent of more elaborately designed LV leads, better delivery equipment, and more skilled operators [13]. However, the endocardial approach can still be challenging due to difficulties with coronary sinus access and lack of suitable implant sites, including no suitable vessels, high threshold, PNS, and lead instability. In cases in which endocardial LV lead implantation is not feasible, surgical placement of epicardial LV leads can be used. Several prospective, randomized trials and retrospective studies have demonstrated that epicardial LV lead placement is not inferior to endocardial LV lead placement for echocardiographic and clinical outcomes, with excellent long-term safety outcomes [14,15,16]. However, most of the previous data were derived from patients treated with sternotomy or thoracotomy, which is a more invasive treatment than thoracoscopic surgery [9,10,17]. A small number of studies have been conducted on the performance of LV lead implanted epicardially using thoracoscopy. However, the efficacy of CRT using the epicardial thoracoscopic approach was not compared with that using endocardial LV leads; if ever compared, only for a short-term follow-up period(6–12 months) [18,19].

In contrast, our data revealed that the VATS epicardial LV lead implantation without sternotomy showed satisfactory and comparable post-discharge (34.3 ± 28.6 months) outcomes with regard to LV lead performance, electro-/echocardiographic improvement, and clinical prognosis of patients, compared with those using a conventional endocardial approach. Our results are in line with data from Marini et al., who demonstrated no differences in death, cardiovascular hospitalizations, or device-related complications between the Epicardial (thoracoscopically implanted) and Endocardial groups during a median follow-up of 24 months [19].

Appropriate LV lead placement into the optimal pacing site is important for the correction of LV dyssynchrony and leads to better clinical outcomes of CRT [20,21,22]. Epicardial LV lead implantation through an open chest or thoracoscopy technique offers direct visual control and allows an easier approach to the optimal pacing site overcoming unfavorable cardiac vein anatomy. However, the open-chest or thoracostomy (standard or minimally invasive) approach is associated with more post-operative pain, larger surgical scar, and longer hospital stay for recovery, compared with the VATS approach. Accordingly, VATS epicardial LV lead implantation can be a better option than full sternotomy or standard thoracotomy.

There are several concerns about the implantation of epicardial LV leads using VATS. First, many physicians may be reluctant to refer patients for this surgical treatment because general anesthesia with one-lung ventilation itself can aggravate patients with severe LV systolic dysfunction. Second, extensive experience of operators is required for this thoracoscopic technique. Finally, if the VATS approach fails, total operation time may be more prolonged with median sternotomy or left thoracotomy eventually required. However, general anesthesia with one-lung ventilation was well tolerated by all of our patients with severe LV systolic dysfunction (range of LVEF, 18 to 35%). Additionally, pacing threshold and impedance were found to be stable and within a clinically acceptable range over the entire follow-up period in the VATS epicardial LV lead group. Ceresa et al. also found that the screw-in of the epicardial LV lead through a small mini-thoracotomy ensures a low pacing threshold and stable impedance both in early and medium terms, supporting our results [23]. No significant in-hospital and post-discharge complications were observed in the Epicardial group. Similarly, in previous studies, the VATS approach was associated with reduced surgical-related complications [10,19,24,25]. In terms of lead dislodgement, the epicardial implant can have a definite advantage over the endocardial approach. Indeed, late LV lead dislodgement occurred only in the Endocardial group. Therefore, epicardial LV lead implantation using VATS can be effectively and safely used as a rescue method for patients with recurrent LV lead dislodgement, or even as a de novo way for those with unfavorable cardiac vein anatomy or when better targeted LV pacing is required.

One of the major limitations of this study is the retrospective, observational design of the analysis. Second, small numbers of patients in the single-center in the Epicardial LV lead group may produce bias; thus, prospective randomized studies on a larger scale are needed to validate the efficacy and safety of the LV lead implant using VATS for CRT more definitely. Finally, only screw-in unipolar pacing leads were used in this study. Therefore, current results cannot be generalized and compared with bipolar systems. However, Ceresa et al. have already shown the stable and satisfactory performance of bipolar pacing leads in their own data [23].

## 5. Conclusions

Where suitably trained thoracoscopic surgeons are available, epicardial LV lead implantation using VATS can be an effective and safe alternative to the standard transvenous approach in CRT patients with difficult vein anatomy or endocardial lead dislodgement. The VATS epicardial LV leads implantation showed stable acute and post-discharge performance, offering comparable CRT outcomes to those observed in patients treated using endocardial LV leads.

## Figures and Tables

**Table 1:** Detailed information on patients with epicardial left ventricular lead.

No.	Age (years)	Sex	Etiology of Heart Failure	NYHA	QRSd (ms)	QRS Morphology	LVEF (%)	Previous Surgery	History of Cardiac Surgery	Surgical Technique
1	49	F	Non-ICMP	III	224	LBBB	17.7		Absence of suitable lateral cardiac vein branches	Thoracoscopy
2	45	M	Non-ICMP	III	184	LBBB	24.7		Absence of suitable lateral cardiac vein branches	Thoracoscopy
3	62	M	Non-ICMP	III	214	LBBB	23.0	AVR	Tortuous target branches	Thoracoscopy
4	62	M	ICMP	II	168	LBBB	26.3	CABG, MVR	Absence of suitable lateral cardiac vein branches	Thoracoscopy
5	70	F	Non-ICMP	II	208	LBBB	23.3		Recurrent LV lead dislodgement & PNS	Thoracoscopy
6	73	M	ICMP	III	182	LBBB	24.3	CABG	Recurrent LV lead dislodgement & PNS	Thoracoscopy with mini-thoracotomy
7	64	M	Non-ICMP	IV	194	LBBB	23.4	MVR	Anomalous cardiac veins	Thoracoscopy
8	53	M	Non-ICMP	III	214	RBBB	25.0		Tortuous coronary sinus and target branches	Thoracoscopy with mini-thoracotomy
9	70	F	Non-ICMP	III	250	LBBB	30.0		Absence of suitable lateral cardiac vein branches	Thoracoscopy with mini-thoracotomy
10	61	F	Non-ICMP	III	166	LBBB	32.0		Absence of suitable lateral cardiac vein branches	Thoracoscopy
11	62	M	ICMP	II	145	LBBB	30.1	CABG	Coronary sinus atresia with unroofed coronary sinus	Thoracoscopy with mini-thoracotomy
12	74	F	Non-ICMP	II	168	LBBB	28.4		Tortuous coronary sinus and target branches	Thoracoscopy
13	59	F	Non-ICMP	II	151	LBBB	35.0	VSD closure	Anomalous cardiac veins	Thoracoscopy with mini-thoracotomy

AVR = aortic valve replacement, CABG = coronary artery bypass graft; ICMP = ischemic cardiomyopathy, LV = left ventricle, LVEF = left ventricular ejection fraction, MVR = mitral valve replacement, NYHA = New York Heart Association functional classification, QRSd = QRS duration, VSD = ventricular septal defect, LBBB = left bundle branch block, RBBB = right bundle branch block, PNS = phrenic nerve stimulation.

**Table 2:** Baseline characteristics.

	Epicardial LV Lead (*n* = 13)	Endocardial LV Lead (*n* = 243)	*p*-Value
Age, years	61.9 ± 8.9	66.2 ± 12.7	0.225
Male gender	6 (46.2%)	152 (62.6%)	0.236
BMI (kg/m^2^)	24.2 ± 2.9	23.3 ± 3.7	0.318
Hypertension	6 (46.2%)	134 (55.1%)	0.526
Diabetes	4 (30.8%)	89 (36.6%)	0.669
Chronic kidney disease	1 (7.7%)	33 (13.6%)	0.542
Stroke	3 (23.1%)	23 (9.5%)	0.113
Previous PCI	1 (7.7%)	47 (19.4%)	0.294
Previous cardiac surgery	5 (38.5%)	42 (17.3%)	0.055
Ischemic cardiomyopathy	4 (30.8%)	65 (26.7%)	0.750
LBBB	12 (92.3%)	210 (86.4%)	0.542
QRS duration (msec)	190 ± 31	170 ± 36	**0.019**
LVEF (%)	26.4 ± 4.6	25.6 ± 6.2	0.701
LVEDV (ml)	230 ± 46	233 ± 77	0.848
LVESV (ml)	181 ± 55	175 ± 66	0.615
CRT-defibrillator	12 (92.3%)	233 (95.9%)	0.538
Lateral LV pacing site	13 (100%)	227 (93.4%)	0.339
Non-apical LV pacing sites	13 (100%)	242 (99.6%)	0.817
LV pacing threshold (V)	1.5 ± 1.0	1.3 ± 0.8	0.651
LV pacing pulse width (ms)	0.5 ± 0.1	0.5 ± 0.1	0.538
LV lead impedance (Ω)	354 ± 51	673 ± 267	**<0.001**
***Discharge Medications***			
Beta-blocker	9 (69.2%)	181 (74.5%)	0.673
ARB/ACEi	11 (84.6%)	20 (84.4%)	0.980
Spironolactone	9 (69.2%)	178 (73.3%)	0.750

Values are expressed as *n* (%) or mean ± SD. LV = left ventricle, BMI = body mass index, PCI = percutaneous coronary artery intervention, ARB = angiotensin receptor blocker, ACEi = angiotensin converting enzyme inhibitor, LBBB = left bundle branch block, LVEF = left ventricular ejection fraction, LVEDV = left ventricular end-diastolic volume, LVESV = left ventricular end-systolic volume, CRT = cardiac resynchronization therapy.

**Table 3:** A comparison of the two groups at baseline and during the follow-up.

	Epicardial LV Lead (*n* = 13)			Endocardial LV Lead (*n* = 243)			*p* Value
	Baseline	Last Follow-Up	*p* Value *	Baseline	Last Follow-Up	*p* Value *	
*Follow-Up*							
Follow-up duration, month	34.3 ± 28.6			37.1 ± 32.7			0.913
BiV pacing percentage (%)	96 ± 7			94 ± 16			0.772
***LV Lead Performance***							
Threshold (V)	1.5 ± 1.0	1.7 ± 0.7	0.154	1.3 ± 0.8	1.5 ± 1.0	**<0.001**	0.365 †
Pulse width (ms)	0.5 ± 0.1	0.5 ± 0.2	0.168	0.5 ± 0.1	0.5 ± 0.1	0.433	0.500 †
Impedance (Ω)	354 ± 51	349 ± 62	0.859	673 ± 267	694 ± 309	0.231	0.483 †
***Electrocardiographic Outcomes***							
QRS duration (ms)	190 ± 31	150 ± 31	**<0.001**	170 ± 36	141 ± 22	**<0.001**	0.602 †
***Echocardiographic Outcomes***							
LVEF (%)	25.7 ± 3.9	37.7 ± 15.1	**0.023**	25.6 ± 6.2	38.1 ± 14.5	**<0.001**	0.866 †
LVEDV (ml)	218 ± 44	192 ± 83	0.484	236 ± 76	192 ± 91	**<0.001**	0.592 †
LVESV (ml)	174 ± 60	118 ± 60	**0.036**	177 ± 64	132 ± 82	**<0.001**	0.768 †

Values are expressed as *n* (%) or mean ± SD. *, comparison between the baseline and the last follow-up values. †, comparison of the relative changes in variables ((baseline-last FU)/baseline × 100) between the Epicardial and Endocardial groups. LV = left ventricle, CRT = cardiac resynchronization therapy, BiV = biventricular, LVEF = left ventricular ejection fraction, LVEDV = left ventricular end-diastolic volume, LVESV = left ventricular end-systolic volume.

## Data Availability

All data generated or analyzed during this study are included in this article. Further inquiries can be directed to the corresponding author.

## Supplementary Materials

The following supporting information can be downloaded at: https://www.mdpi.com/article/10.3390/jcdd9050160/s1, Table S1. Details of a comparison of the two groups at baseline and during 6- and 12-month Follow-up.
