# Supplementary material for: Thoracoscopic Implantation of Epicardial Left Ventricular Lead for Cardiac Resynchronization Therapy
Source: J Cardiovasc Dev Dis. 2022 May 16;9(5):160. doi: 10.3390/jcdd9050160 (PMC9145737; doi:10.3390/jcdd9050160)
Supplement: Supplementary file 1 [file jcdd-09-00160-s001.zip › jcdd-1703710-supplementary.pdf]

Thoracoscopic implantation of epicardial left ventricular lead for cardiac resynchronization therapy

Hye Ree Kim, et al.

Supplementary materials

Table S1. Details of a comparison of the two groups at baseline and during 6 and 12-month Follow-up

Parameter	Epicardial LV lead (n=13)			Endocardial LV lead (n=243)			*p*-value*
	Basal	6 month	*p*-value	Basal	6 month	*p*-value	
Threshold, V	1.5±1.0	1.8±0.9	0.055	1.3±0.8	1.4±1.0	0.012	0.214
Impedance, Ω	354±51	363±87	0.938	673±267	713±285	0.065	0.631
	Basal	12 month	*p*-value	Basal	12 month	*p*-value	
Threshold, V	1.5±1.0	1.7±0.9	0.225	1.3±0.8	1.5±1.1	<0.001	0.427
Impedance, Ω	354±51	388±90	0.173	673±267	717±312	0.064	0.666

*, comparison of the relative changes in variables [(baseline – 6month or 12month) / baseline x 100] between the Epicardial and Endocardial groups.

LV = left ventricle
